# Clinical, Parasitological, and Serological Follow-Up of Dogs with Sarcoptic Mange Treated Orally with Lotilaner

**DOI:** 10.1155/2021/6639017

**Published:** 2021-01-25

**Authors:** F. Moog, J. Brun, P. Bourdeau, M. C. Cadiergues

**Affiliations:** ^1^Small Animal Clinic, Université de Toulouse, ENVT, Toulouse, France; ^2^LUNAM, University-ONIRIS-DPMA Unit/NP3 Unit, Nantes, France; ^3^INFINITY, Université de Toulouse, INSERM, CNRS, UPS, Toulouse, France

## Abstract

Canine sarcoptic mange is a highly pruritic and contagious skin disease caused by the mite *Sarcoptes scabiei* var. *canis*. This case series describes the clinical, parasitological, and serological follow-up of a cohort of eight adult Saint Bernard dogs with confirmed sarcoptic mange, treated orally with lotilaner. Dogs were evaluated initially and after 14 days and 1, 2, 3, 4, 6, and 12 months for skin lesions, pruritus severity, presence of parasites, and *Sarcoptes*-IgG levels. A serological indoor allergy panel (IgE) was obtained for seven dogs at day 0 and repeated 12 months later in five dogs to assess potential cross-reactivity between *S. scabiei* and environmental allergens. Lotilaner was administered to each dog according to the manufacturer's instructions and was repeated after one and two months without any concurrent therapeutic measure or modification of the husbandry conditions. Pruritus ceased after two weeks. The cutaneous score was reduced by 47%, and skin scrapings were negative for all but three animals. All skin scrapings were negative after one month. Lesions were absent after two months. Serological levels decreased gradually, but more slowly than the skin lesions, and two dogs out of six remained positive in the absence of skin lesions or symptoms. All dogs initially tested positive for dust mites and/or storage mites. The IgE titres remained unchanged 12 months later in the five tested dogs. This case report demonstrates the efficacy of lotilaner on scabies in a cohort of infested dogs under natural conditions and the potential antigenic cross-reaction of *S. scabiei* with house dust and storage mites.

## 1. Introduction

Canine sarcoptic mange is a highly pruritic and contagious skin disease caused by the mite *Sarcoptes scabiei* var. *canis* [1]. Diagnosis is ideally confirmed by multiple superficial skin scrapings showing the presence of mites, positive serology [2], and/or response to a specific therapy. Cross-sensitisation with house dust mites has been documented [3, 4]. Treatment is based on administration of an acaricide to the affected dog and all canine in-contacts. Amongst the different groups of acaricidals, the recently developed isoxazolines have produced good results [5–9].

This case series describes the clinical, parasitological, and serological follow-up of a cohort of nine adult Saint Bernard dogs with sarcoptic mange, treated orally with lotilaner.

## 2. Case Description

Nine adult Saint Bernard dogs living in the south of France and belonging to the same owner were referred for intense pruritus of several weeks' duration. The animals were part of a professional breeding program and were born and raised on the property, in a rural environment. They all lived in the same enclosure, with built-in concrete semi-individual housing and a natural grass exercise area.

The mean age of the dogs was 4.3 years (range 2-7). The cohort included 2 males and 7 females, all intact. The mean weight was 55.3 kg with individual weights below their normal values [10] as a result of weight loss since the onset of clinical signs (Table 1). A thorough clinical history was obtained from the owner and referring veterinarian. The dermatological issue had started four months earlier, with one dog initially affected. Prior to this episode, none of the dogs had ever presented any skin problem. The main sign reported by the owner was pruritus localised on the ears and flanks. This was followed by focal alopecia, with crusts and scaling observed on and around the pruritic areas. Other animals became affected within a few days, and after a week, all animals exhibited similar clinical signs. All the dogs were treated with antiseptic/antifungal shampoos (Malaseb®, Dechra veterinary products), and some of them received a systemic antibiotic therapy (amoxicillin/clavulanic acid 15 mg/kg twice daily orally) for about three weeks without success and were then referred for further investigation. On presentation, all dogs, although underweight, were otherwise healthy. All general physical parameters were normal.

**Figure 1:**
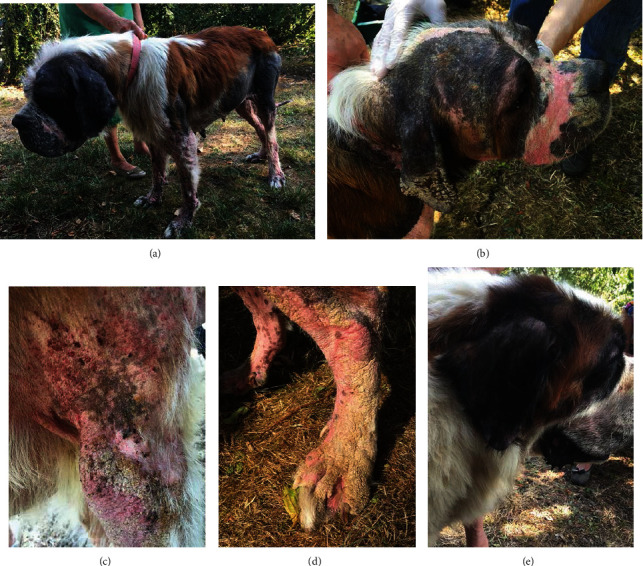
Initial physical examination. Extensive alopecia and poor body condition (a); close view of the head: alopecia, erythema, crusts, and scaling localised mainly on the convex aspect of the pinna and muzzle (b). Self-inflicted lesions associated with alopecia, papules, and thick crusts on the shoulder and elbow (c). Alopecia, erythema, and thick scaling/crusting on the distal part of a hind leg (d). Least-affected dogs only showed alopecia on the pinnae and face (e).

Dermatological examination revealed similar cutaneous signs on all animals, albeit at different levels of severity. Signs included regional alopecia, diffuse erythema, papules, crusts, and scaling localised mainly on the convex aspect of the pinnae, lateral aspect of the elbows, ventral and lateral thorax, limbs, and abdomen (Figure 1).

Due to the sudden onset of pruritus with a progressive number of animals affected along with the clinical signs and lesion pattern, sarcoptic mange was highly suspected. On further questioning, the owner revealed that, a few weeks earlier, a dead red fox had been found lying against the dogs' metal enclosure and that the dogs had been in contact with the body through the small holes in the fence. The owner also mentioned that she had been intensively pruritic herself since shampooing the dogs.

Superficial skin scrapings were obtained from the affected areas and revealed the presence of numerous live *S. scabiei* mites on all dogs.

Lotilaner (Credelio®, Elanco GmbH) was selected as the sole acaricidal treatment [11], based on the activity of the isoxazoline group against sarcoptic mange [5–9]. As lotilaner is not specifically licensed for sarcoptic mange treatment, written consent was obtained from the dogs' owner prior to beginning the therapeutic procedure and follow-up.

The appropriate dose of lotilaner was administered to each dog by the owner, according to the manufacturer's label prescription, to attain the recommended dose of 20-43 mg/kg. A suitable combination of 450 mg tablets was administered to each dog to ensure an average dose of lotilaner of 24.5 mg/kg. Second and third courses of treatment were given by the owner one and two months later, according to the manufacturer's recommendations. Thus, each animal was treated three times at one-month intervals. No concurrent treatment or modification of husbandry conditions was implemented.

### 2.1. Follow-Up Schedule

Four parameters were used to monitor the dogs: scoring of symptoms and lesions, microscopical search of mites on skin scrapings, and serology tests to quantify IgG antibodies against *S. scabiei*. The dogs were examined eight times, initially before the administration of lotilaner (day 0) and after two weeks and 1, 2, 3, 4, and 6 months.

A previously used cutaneous scoring system [8] was adopted, based on seven criteria (alopecia, crusts, papules, pustules, scaling, lichenification, and erythema) scored out of 6 (0 = no lesion, 1 = mild, 3 = moderate, and 6 = severe) over 14 body regions (periocular region, head, neck, ears, sternum, chest, flanks, abdomen, tail, perineum, back, axilla, and thoracic and pelvic limbs). The maximum score was 588. Superficial skin scrapes were obtained from four areas of lesions on each dog and examined microscopically by the same trained dermatologist. Pruritus was assessed by the owner using a modified visual analogue scale out of 10 [12]. Serological testing was performed using a commercial immunoassay (Sarcoptes-ELISA 2001®, AFOSA GmbH) on day 0 and subsequently 2, 3, 4, and 6 months later.

A serological allergy panel (IgE) against 11 indoor allergens was obtained on day 0 (GREER® Aller-g-complete®, Idexx, France) to assess potential cross-reactivity between *S. scabiei* and other common canine aeroallergens.

A final examination was implemented one year later when all parameters except skin scrapings were evaluated.

### 2.2. Results after Treatment (Figures 2–4)

On day 0, the mean dermatological score was 174.9 ± 66 out of 558 (min 91, max 257). At least one area on each dog was positive for either adult or immature stages of *Sarcoptes* mites or eggs, and the average level of pruritus was 6.82 out of 10. IgG levels against *S. scabiei* tested strongly positive against the reference value (mean 73%, min 40.6%, max 112%; reference positive value: 20%).

Two weeks later, the cutaneous score was 92.5 on average (min 37, max 146), corresponding to a decrease of 47% as compared to day 0. Skin scrapings were negative for all but three animals. Three dead adult mites were found on two dogs, and a single egg was observed on the third dog. The pruritus score was nil and subsequently remained as such.

One month after the first dosing, all skin scrapings were negative (eight dogs). When the dogs were reexamined 2, 3, 4, and 6 months after the initial treatment, no active lesions could be found and so no skin scrapings were performed. A decrease in serology levels was recorded over time with an average value of 38.5% after 2 months, 27.3% at 3 months, and 21.8% at 6 months. This corresponded to a 47% reduction rate for month 2, a 63% reduction rate for month 3, and 70% for month 6 compared to day 0. However, a mild transient rise in serology levels was observed in three dogs for month 4 (37.3% positive value) compared to month 2. The cutaneous score also decreased steadily and regularly with a score of 31.1 after 1 month, 13.6 at 2 months, 1.1 at 4 months, and 0 after 6 months which corresponded to a decrease of 82%, 85%, 99%, and 100% compared to day 0, respectively.

**Figure 4:**
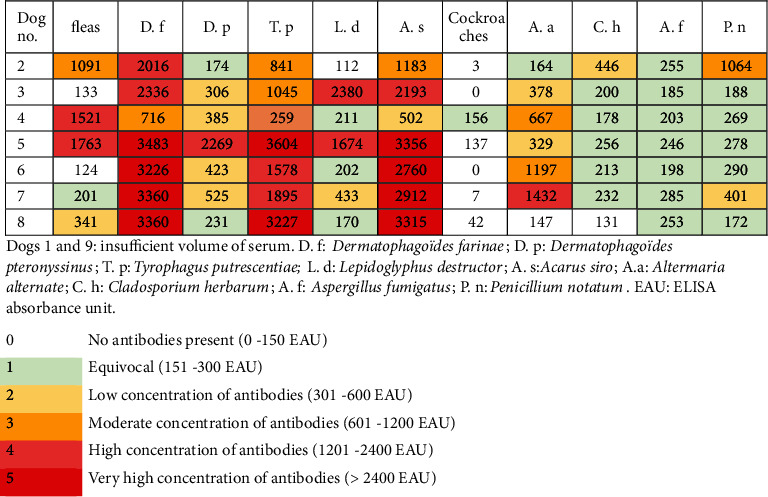
Specific allergen IgE serology (indoor panel) results on initial presentation.

The allergy panel yielded results for 7 of the 9 dogs. The serum of the remaining two dogs could not be tested due to technical issues (Figure 4). The results showed that 4 of these 7 dogs tested very strongly positive (>2400 EAU) against *Dermatophagoides farinae* and *Acarus siro* whereas the other three dogs tested either moderately (601-1200 EAU) or strongly positive (1201-2400 EAU) against these mites. Against *Tyrophagus putrescentiae*, two dogs tested very strongly positive, two dogs strongly positive, and two dogs moderately positive. Against *D. pteronyssinus*, one dog tested strongly positive, four dogs weakly positive (301-600 EAU), and two dogs were borderline (151-300 EAU). Against *Lepidoglyphus destructor*, two dogs tested moderately positive, one weakly positive, three borderline, and one below the threshold. Fleas gave mixed results with two dogs testing strongly positive, one dog weakly positive, one dog borderline, and one dog below the threshold. As expected, the results against moulds remained low or borderline, except for *Alternaria alternata*, for which one dog tested strongly positive, two dogs moderately positive, two dogs weakly positive, and two dogs borderline or below the threshold.

The weights of all animals increased steadily during the treatment, eventually attaining the values recorded before disease development.

No adverse effect was reported or observed that could be attributed to the administered product.

Dog #1 died following gastric dilatation volvulus three weeks after initial presentation.

### 2.3. Final Examination at 12 Months (Figure 5)

**Figure 5:**
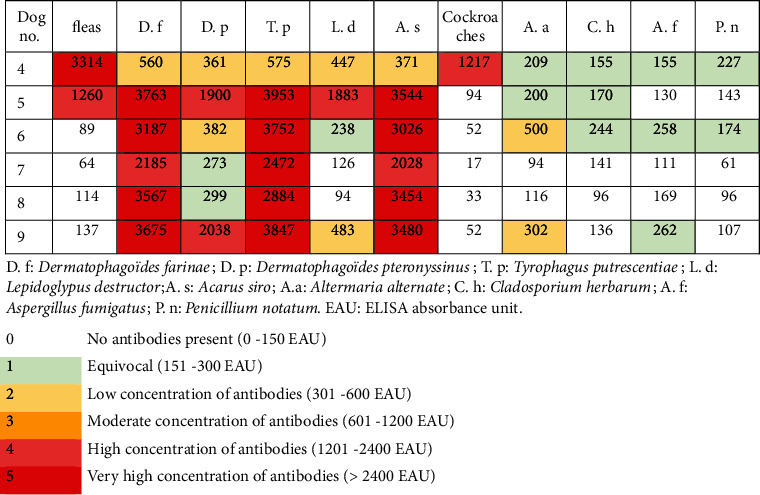
Specific allergen IgE serology (indoor panel) results 12 months after initial presentation.

By twelve months after the initial presentation, dogs #2 and #3 had been euthanized due to complications following caesarean section and acute renal failure, respectively. No further antiparasitic treatment was suggested after the initial three months. Dog # 8 had a pruritus score of 5/10 due to pyotraumatic dermatitis likely due to mechanical trauma, whereas all the other dogs had a 0/10 pruritus score. The average cutaneous score was 1.6 out of 558, and the average IgG level against *S. scabiei* was 24.8% (13 to 45%). Dogs #4 and #5 still had relatively high values (41 and 45%, respectively) without displaying any pruritus or lesions from month 3 to month 12. Dogs #2 and #3 had tested negative during their last control at six months. Specific IgE titres remained relatively unchanged 12 months later in the five dogs previously tested (Figure 5).

## 3. Discussion

The clinical signs, in this cohort of dogs affected by sarcoptic mange, were compatible with those described in the literature [1, 13–17]. It is likely that direct contact with the deceased red fox had initiated the process as *S. scabiei* are endemic in red foxes in Europe and can act as a source of infestation for domestic animals [18, 19]. The highly contagious aspect of the disease was clearly apparent with all the animals progressively and rapidly becoming affected, as well as the transmission to humans with the development of transient cutaneous signs on the owner [1, 20].

Affected dogs are reported to show seroconversion 3 to 5 weeks after the initial infestation and 1 to 3 weeks after the development of clinical signs [21]. The serological values are gradually reduced by progressive elimination of the antibodies present and the cessation of new synthesis. This indicates a real disappearance of antigenic stimulation, and thus of the actual parasites, that the negative values of the scrapings are unable to demonstrate. The persistence of positive values in two dogs despite the absence of symptoms and lesions remains unusual although antibodies can be maintained for several months [2, 22] and up to 6 months in the laboratory according to one of the authors (PB). Interestingly, the three dogs with the highest initial ELISA values were also those with a persistently high clinical score and a higher titre than the positive value of 20-25%, at the one-year evaluation. This underlines the importance of performing the serological test before and after the treatment to obtain a kinetic evaluation. A single isolated test after the treatment cannot be interpreted unless the values are clearly negative.

Previous studies have suggested that a hypersensitivity reaction is involved in the pathogenesis of scabies as the pruritus is out of proportion to the low number of mites, and the superficial eosinophilic and mastocytic perivascular dermatitis present in sarcoptic mange is similar to many hypersensitivity-mediated dermatoses [1, 22, 23].

Cross-antigenicity between *S. scabiei* and the dust mite *D. farinae* has been demonstrated in dogs and humans. Animals affected by sarcoptic mange test positive for *D. farinae* either by intradermal or serological tests [22, 23]. This was confirmed in the present cohort with four dogs reacting very strongly, two dogs strongly, and one moderately to this house dust mite. *D. farinae* is commonly involved in canine atopic dermatitis [24] and gave the highest number of positive reactions in atopic dogs in one study [25]. However, it is also important to note that a high percentage of healthy dogs can demonstrate hypersensitivity reactions when tested [26].

Cross-reactivity has been thought to be higher between *D. farinae* and *D. pteronyssinus* than between storage and house dust mites [27]. However, in this cohort, the mean serology levels were higher for *D. farinae*, *Tyrophagus putrescentiae*, and *Acarus siro* than for *D. pteronyssinus.* This correlates partially with a previous *in vitro* study that demonstrated extensive cross-reactivity not only between *D. farinae* and the storage mites *T. putrescentiae* and *A. siro* but also between *D. farinae* and *D. pteronyssinus* [27].

Despite this cross-reactivity, the sensitisation to dust mites caused by sarcoptic mange does not seem to lead to the development of atopic dermatitis [23]. In this cohort of dogs, none had a history of skin problem prior to this episode and all animals returned to normal and never developed signs of atopic dermatitis. The serology levels for moulds and fungi remained low.

One limit of this report is that dust mite-specific IgE have been shown to be naturally present in a high number of nonatopic dogs [28]. Due to the nature of this case report, circulating antibodies could not be measured before the development of sarcoptic mange, and the cross-sensitisation and low level of circulating antibodies to *S. scabiei* and dust mites may already have been present. However, it has been shown experimentally that these antibody levels, even if present initially, became much higher following infestation by mite [22]. This observation reinforces the need for the practitioner to adopt a rigorous diagnostic approach in pruritic dogs. Atopic dermatitis diagnosis is strictly clinical and requires the elimination of other causes of pruritus, particularly parasites and infections. The allergological investigation is only undertaken as a second step [29].

Interestingly, a transient rise in *S. scabiei* antibodies was observed four months after the initial treatment. A similar observation had already been reported, and it was hypothesized that the mites induced the greatest amount of antigens while they were decomposing in the skin [22].

Due to the low sensitivity of the skin scraping technique, a few mites or eggs may have been missed at the one-month examination, and it was decided to continue the administration of lotilaner at monthly intervals as a licensed flea and tick preventative [11].

Despite the small number of dogs, the results of this study are consistent with reports describing the efficacy of other isoxazoline molecules such as afoxolaner [5], fluralaner [6, 8], and sarolaner [7] against *S. scabiei* in dogs. All three molecules are registered in several countries for the treatment of sarcoptic mange in dogs. Lotilaner has previously been shown to be an effective and safe treatment against fleas and ticks [30–34] and is licensed solely for that purpose. However, it has already been demonstrated to possess broader potent acaricidal properties against mites such as *Demodex canis* [35]. As a hypersensitivity reaction is involved in the mechanisms of pruritus induction in sarcoptic mange, the presence of dead mites after treatment may cause pruritus in dogs several weeks after parasitological cure [1, 2, 36]. The fact that no living mite was found after two weeks and that no pruritus was detected at that time in any of the dogs suggests that lotilaner exhibits rapid miticidal activity.

In summary, this report describes a cohort of adult dogs from the same household naturally affected by sarcoptic mange. The serology correlated with the clinical diagnosis and partial cross-sensitisation with house and storage mites was observed, although all dogs could not be tested. The novel isoxazoline lotilaner was used at monthly intervals to treat the condition and confirmed its broad acaricidal properties by allowing a rapid and effective elimination of the mites and resolution of clinical signs.

## Figures and Tables

**Figure 2:**
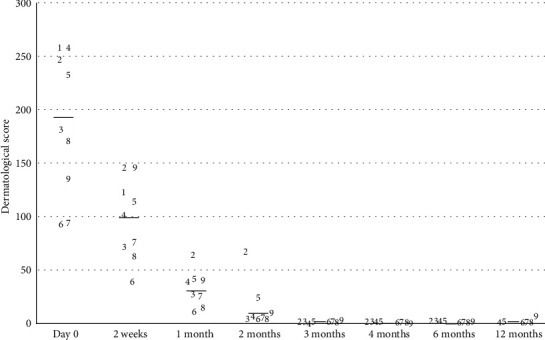
Progression of the dermatological score for the dogs over time after treatment with lotilaner on day 0, 1 month, and 2 months. Each dog is represented by its number (1-9). The black line represents the average value.

**Figure 3:**
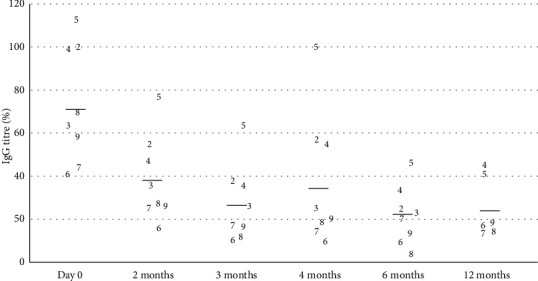
Progression of the *Sarcoptes*-IgG titre of the dogs over time after treatment with lotilaner on day 0, 1 month, and 2 months. Each dog is represented by its number (1-9). The black line represents the average value.

**Table 1:** Description of the canine cohort on initial presentation.

Dog no.	Gender	Age (years)	Weight (kg)	Body condition score [10]	DS	PS	IgG titre (%)
1	F	3	49.3	2/9	257	8.2	
2	F	3	38.8	2/9	247	8.1	100
3	M	6	60.0	3/9	178	3.9	63.4
4	F	2	47.7	2/9	256	9.1	98.9
5	F	7	51.0	4/9	231	6.0	112
6	F	6	46.0	3/9	91	2.0	40.6
7	F	4	63.7	4/9	92	8.3	43.4
8	M	4	71.2	4/9	169	8.0	69.5
9	F	4	69.8	5/9	135	9.2	57.1

F: female; M: male; DS: dermatological score; PS: pruritus score.

## Data Availability

The data generated and/or used during the workup of this case series cannot be made publicly available in the interests of retaining patient confidentiality but are available from the corresponding author on reasonable request.
